# Differences in velopharyngeal pressures during speech sound production in patients with unilateral cleft lip and palate (UCLP) and healthy individuals

**DOI:** 10.3205/000328

**Published:** 2024-03-01

**Authors:** Simone Miller, Johanna Kallusky, Rüdiger Zimmerer, Frank Tavassol, Nils-Claudius Gellrich, Martin Ptok, Michael Jungheim

**Affiliations:** 1 Department of Phoniatrics and Pediatric Audiology of the Department of Otolaryngology, Hannover Medical School, Hanover, Germany; 2 Institute of General Practice and Palliative Care, Hannover Medical School, Hanover, Germany; 3 Department of Oral and Maxillifacial Surgery, University Hospital Tübingen, Germany; 4 Department of Oral and Maxillofacial Surgery, University Medicine Halle, Germany; 5 Department of Oral and Maxillofacial Surgery, Hannover Medical School, Hanover, Germany; 6 Department of Phoniatrics and Pediatric Audiology, Hannover Medical School, Hanover, Germany; 7 HNO Phoniatrie Praxis, Bremen, Germany

**Keywords:** nonsyndromic cleft, velopharyngeal insufficiency, high resolution manometry, articulation, cleft lip and palate, velopharyngeal function

## Abstract

**Background:**

During articulation the velopharynx needs to be opened and closed rapidly and a tight closure is needed. Based on the hypothesis that patients with cleft lip and palate (CLP) produce lower pressures in the velopharynx than healthy individuals, this study compared pressure profiles of the velopharyngeal closure during articulation of different sounds between healthy participants and patients with surgically closed unilateral CLP (UCLP) using high resolution manometry (HRM).

**Materials and methods:**

Ten healthy adult volunteers (group 1: 20–25.5 years) and ten patients with a non-syndromic surgically reconstructed UCLP (group 2: 19.1–26.9 years) were included in this study. Pressure profiles during the articulation of four sounds (/i:/, /s/, /ʃ/ and /n/) were measured by HRM. Maximum, minimum and average pressures, time intervals as well as detection of a previously described 3-phase-model were compared.

**Results:**

Both groups presented with similar pressure curves for each phoneme with regards to the phases described and pressure peaks, but differed in total pressures. An exception was noted for the sound /i:/, where a 3-phase-model could not be seen for most patients with UCLP. Differences in velopharynx pressures of 50% and more were found between the two groups. Maximum and average pressures in the production of the alveolar fricative reached statistical significance.

**Conclusions:**

It can be concluded that velopharyngeal pressures of patients with UCLP are not sufficient to eliminate nasal resonance or turbulence during articulation, especially for more complex sounds. These results support a general understanding of hypernasality during speech implying a (relative) velopharyngeal insufficiency.

## Zusammenfassung

**Hintergrund:**

Während der Artikulation öffnet und schließt sich der velopharyngeale Abschluss in schnellen Bewegungsabfolgen und für die Bildung einiger Laute ist ein vollständiger Abschluss notwendig. Für Patient_innen mit Lippen-, Kiefer-, Gaumenspalte (LKG) ist bekannt, dass die Verschlussdrücke im Velopharynx beim Schlucken reduziert sind. Ziel dieser Studie war es, die Druckprofile von Patient_innen mit unilateraler LKG und gesunden Proband_innen mittels Hochauflösungsmanometrie (HRM) zu vergleichen und zu evaluieren, ob bei der Produktion verschiedener Sprachlaute ebenfalls Druckunterschiede im Velopharynx bestehen.

**Material und Methoden:**

Es wurden 10 gesunde Erwachsene (Gruppe 1: 20–25,5 Jahre) und 10 Patient_innen mit einer nicht-syndromalen operativ verschlossenen unilateralen LKG (Gruppe 2: 19,1–26,9 Jahre) untersucht. HRM-Verschlussdruckprofile wurden während der Artikulation von 4 Sprachlauten (/i:/, /s/, /ʃ/ und /n/) aufgezeichnet. Es wurden Maximal-, Minimal- und Durchschnittsdrücke sowie Zeitintervalle ermittelt. Zudem wurden die Druckprofile mit einem zuvor beschriebenen 3-Phasen-Phonationsmodell verglichen.

**Ergebnisse:**

In beiden Gruppen zeigten sich in Bezug auf die Phonationsphasen ähnliche Druckprofile für die produzierten Laute, allerdings lagen unterschiedliche Gesamtdrücke vor. Eine Ausnahme stellte die Realisation des Lautes /i:/ dar, bei der sich bei den meisten Patient_innen mit LKG keine drei Phonationsphasen erkennen ließen. Zwischen den beiden Gruppen existierten Druckunterschiede in der Velopharynxregion von 50% und mehr. Es fanden sich statistisch signifikante Unterschiede bei den Maximaldruck- und durchschnittlichen Druckwerten bei der Produktion von alveolaren Frikativen.

**Diskussion:**

Die reduzierten velopharyngealen Verschlussdrücke bei Patient_innen mit LKG tragen wahrscheinlich dazu bei, dass nasale Resonanzen und Turbulenzen nicht ausreichend eliminiert und komplexere Laute dadurch nicht korrekt gebildet werden. Die hier erhobenen Ergebnisse unterstützen die Annahme, dass die bei Patient_innen mit LKG häufig beobachtete Hypernasalität während der Sprachproduktion auf eine (relative) velopharyngeale Insuffizienz zurückzuführen ist.

## Introduction

During articulation the closure of the velopharynx plays an important role. It results from two different movements of pharyngeal structures [1]. The soft palate elevates towards the posterior pharyngeal wall, which represents the main component of the mechanism. At the same time the lateral pharyngeal walls move medially towards the velum and complete the closure. Since both of the movements are synchronous a sphincter-like closure results [1]. According to Schneider et al. [2] and Witzel et al. [3], there are various types of closures depending on the dominance of one of the two movements and the involvement of the Passavant cushion. This allows for the following classification of the closure mechanism: coronal, circular, circular plus Passavant cushion and sagittal. The closure mechanism is said to vary depending on the sound, the age of the person and between individuals [1]. Impact of the type of closure mechanism on the pressure profiles has not been investigated yet. The closure separates the nasal cavity from the resonance system and thus enables a rise in intraoral pressure to produce fricative and plosive consonants [4]. 

A restricted function of the velopharynx may result in an insufficient closure of the nasal cavity, which creates a nasal sound also for sounds that are not supposed to include nasal resonance. Patients with velopharyngeal insufficiency often feel an impact on the quality of life in communication (orofacial dysfunction [5]). 

In patients with velopharyngeal dysfunction, the group of velopharyngeal insufficiencies due to structural abnormalities mostly due to cleft lip and palate (CLP) is dominating in contrast to velopharyngeal incompetence for neurogenic reasons, for example [6]. Regarding the severe impairments resulting from an open CLP, such as swallowing and sucking disorders, impaired speech [7], facial growth deficiencies [8] and Eustachian tube dysfunction [9], children undergo plastic surgery as well as conservative treatment such as speech and language therapy [10], [11]. Despite the apparent recovery of swallowing functions and absence of nasal regurgitation, adults with a surgically repaired CLP often still experience velopharyngeal insufficiency due to restricted physiological functioning [12], [13], [14]. The muscle function, in particular the sphincter-like closure, cannot be restored surgically and air might continue to leak from the oral into the nasal cavity resulting in hypernasality [15]. 

Indirect procedures for measuring velopharyngeal function during speech, such as digital audio tape recording [16], auditory and spectral analysis [17], as well as nasometry [18], [19], [20], [21], can provide important information about the nasal resonance during articulation, but only allow indirect conclusions or assumptions with regards to velopharyngeal functions. A velopharyngeal closure force sensing bulb has been described to measure the closure force of the velopharynx [22], [23]. Whereas this device might be influencing articulatory movements and the velopharynx function in particular, manometric techniques offer a well-known and tested medical procedure which can also be applied to the velum. By being capable of evaluating the intraluminal pressures of the velopharyngeal closure directly [24], [25], manometric techniques offer a great advantage. Pharyngeal high resolution manometry (HRM), in particular, due to its very small catheter/probe size, has already been reported to be a useful tool investigating pressures during swallowing or speech production [26], [27], [28], [29]. While the contractile reflexes of the upper esophageal sphincter during swallowing and phonation have been investigated [30], [31], [32], [33], [34], velopharynx pressures need to be studied with regards to pressure profiles during articulation [32], [35], [36]. Perera et al. [32], for example, found a phonation-induced pressure increase in the upper esophageal sphincter (UES) in healthy volunteers during phonation tasks using HRM of the UES. A simultaneous videofluoroscopy showed that the larynx did not move dorsally during phonation, which was interpreted as evidence of a reflexive mechanism. As for velopharynx pressures during phoneme production, Jungheim et al. [36] have investigated healthy individuals by HRM and developed a phase model. According to the group [36], the articulation process for sustained phonation of /i:/ shows an initiation, steady state and termination phase. These phases involve an initial pressure increase, a steady state where the pressure stays at a certain level and a final pressure decrease.

Based on the study hypothesis that patients with CLP produce lower pressures in the velopharynx than healthy individuals, as has already been reported by HRM measurement during swallowing [37], this study compared the pressure profiles of the velopharyngeal closure during articulation of different sounds between healthy participants and patients with surgically closed unilateral cleft lip and palate (UCLP) using HRM. It was aimed to evaluate whether the closing pressure profiles during articulation, i.e. the phoneme-specific pressures in the velopharyngeal closure, differ between both groups. From a clinical point of view, it is expected that the results will help to understand velopharyngeal pressure profiles in patients with velopharyngeal insufficiency, particularly those with UCLP and hypernasality. 

## Materials and methods

### Study design

A monocentric prospective experimental study was conducted.

### Volunteers and patients

Ten healthy adult volunteers (group 1: age range: 20–25.5 years; 5 male, 5 female) and ten adult patients with a non-syndromic surgically reconstructed UCLP (group 2: age range: 19.1–26.9 years; 5 male, 5 female) were included in this study. Volunteers were recruited from the medical campus and patients consecutively from the interdisciplinary clinic for orofacial clefts where they regularly underwent routine consultations in the department. Study recruitment aimed for a balanced sex ratio and similar age profile in both the healthy volunteers and patient groups. Inclusion criteria in the patient group were the occurrence of a complete UCLP and hypernasality that had been assessed during routine visits in the department by phoniatricians and speech pathologists and reassessed as part of this study protocol. The exclusion criteria were esophageal diseases, a current pregnancy, oral-nasal fistula, and a history of velopharyngoplasty or different surgery for speech improvements. Older participants above the age of 60 were excluded to account for subclinical changes in the velopharyngeal function that might occur with higher age.

### Selection of phonemes

1. /i:/ describes a front vowel with a high tongue position
2. /s/ describes the voiceless alveolar fricative (alveolar sibilant)
3. /ʃ/ describes the voiceless postalveolar fricative (postalveolar sibilant)
4. /n/ describes the alveolar nasal phoneme

Phonemes of different categories were chosen. The nasal phoneme /n/ does not require any velum activity and therefore is not expected to show any differences among patients and healthy subjects, but acts as a control sound. German vowels require a relatively tight velopharyngeal closure and are generally not said to present with nasality [38]. The sound /i:/ was chosen as a vowel since a previously published model on velopharyngeal pressures during phonation was based on this sound [36]. During the production of fricatives air flows through a narrow channel created by the articulators causing a frication [6]. For this reason, they are considered more complex sounds which also require a tight and coordinated velopharyngeal closure. The two chosen fricatives are also sibilants, which are characterized by an intense high-friction sibilance noise component [17].

### High resolution manometry

HRM was performed as has been described in the literature previously [28], [29], [37], [39]. Data was collected using a solid-state HRM hardware system (Solar GI HRM, Medical Measurement Systems (MMS), Enschede, The Netherlands) with a manometric catheter (Unisensor, Attikon, Switzerland) specifically designed to measure the pharynx and UES. The catheter had an outer diameter of 2 mm and a total of 20 unidirectional pressure sensors of which 19 were spaced at 7.5 mm intervals; and one sensor was located 5 cm distal to the sensors. The catheter was calibrated and sterilized according to the manufacturer’s specifications before each measurement. All pressures were referenced to atmospheric pressure, and data was acquired at a frequency of 50 Hz for each sensor. The collected data was analyzed using MMS software (version 8.20e).

Pressure and time parameter patterns of participants and patients were evaluated in the velopharyngeal area (VP). This region of interest (ROI) was identified in a spatiotemporal plot [24], [40]. During rest, only the upper esophageal sphincter can be identified; the velopharynx and the tongue base show activity during swallowing and/or phonation. Since the velopharynx stretches over an area of around 2 cm, the sensor, which showed the highest pressure, was selected for the analysis. On the basis of the 3-phase-model of Jungheim et al. [36] the act of phonation was manually separated into three phases – the initiation, steady state and termination phase. Data were recorded as follows: 

**Initiation and termination phase**

- Duration of initiation phase: t_1_
- Duration of termination phase: t_3_
- Maximum pressure of initiation phase: P_1max_
- Maximum pressure of termination phase: P_3max_

**Steady state**

- Maximum and minimum pressure: P_2max_ and P_2min_
- Average pressure: P_2Ø_
- Sum of pressure within 3 seconds (area under curve): AUC
- Slope: quotient of the average pressure of the first and last 10% of the duration of the steady state

**Figure 1:**
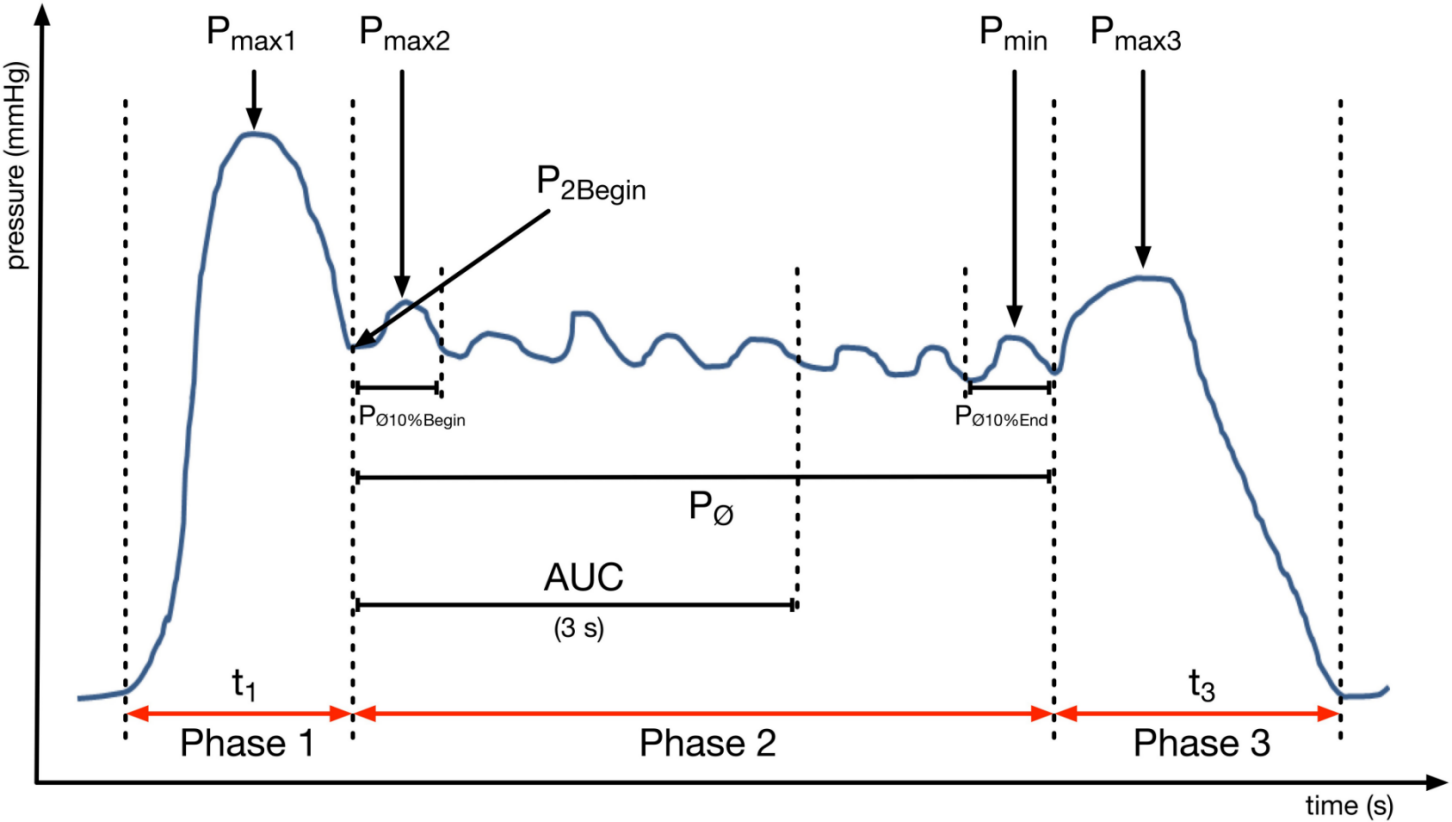
Schematic representation of parameters for analysis of the velopharyngeal pressure curve (in blue) during phonation. P_max_=pressure maximum, P_min_=pressure minimum, t=time, P_Ø_=mean pressure, P_Ø10%_=mean pressure over 10% time frame at either the beginning (begin) or end of phase 2, AUC=area under curve

Most of the parameters (see Figure 1 (Fig. 1)) were provided due to the calculation function of the MMS analysis software and some others were calculated on the basis of these parameters. In order to do so, the pressure curve was visually divided into the three phases and the pressure and time parameters were determined. To calculate the AUC during the steady state, the area under curve was evaluated over a period of three seconds from the beginning of the steady state and then corrected by the AUC during rest.

Regarding the slope, the duration of the steady state was measured first. Within the first and the last 10% of the steady state an average pressure was determined and a quotient calculated to detect a change in pressure.

### Test setting

As described by Kallusky and colleagues [37], a differentiated speech and voice evaluation was performed, including the auditory assessment according to Gutzmann [1], as well as the determination of the vital capacity for the open and closed nasal air passage. All patients were seen during routine visits prior to any study-related procedures, where hypernasality had been diagnosed. During those routine visits, patients undergo several investigations and test procedures, including fibreoptic endoscopy (by a phoniatrician), investigation of the oral and pharyngeal cavity (by a phoniatrician as well as a speech pathologist), direct visual inspection of the velum functions during speech (by a phoniatrician as well as a speech pathologist), auditory sound analysis (by a speech pathologist), vital capacity of the open and closed nasal air passage (by a speech pathologist), A-I test according to Gutzmann (by a speech pathologist), endurance test (during phonation of several sounds, by a speech pathologist), mouth motor activity testing (by a speech pathologist), Czermak-mirror testing for nasal air “discharge” during phonation (by a speech pathologist), as well as a questionnaire regarding quality of life and symptoms.

The HRM catheter was calibrated for body temperature. While the participants and patients with UCLP sat upright with the head in a neutral position, the manometric catheter was placed transnasally into the upper esophagus and fixed in place at the tip of the nose. In patients with UCLP, the nasal side not affected by the cleft was selected for catheter insertion. This side was chosen in an attempt to make measurements more comparable, as due to the cleft as well as the surgery tissue has been modified artificially. To avoid a loss of mucosal sensitivity, lubricating gel containing a local anesthetic agent was not used. The small-diameter catheter passed easily through the nose and was positioned to ensure that the high-pressure area of the UES and the pharyngeal structures above were represented.

Each participant rested for at least 5 min in order to become accustomed to the catheter before performing the experimental tasks. Both healthy volunteers and patients with UCLP were asked to produce the phonemes /i:/, /s/, /ʃ/ and /n/ over a period of 5 seconds each at a volume of 65 dB(A) ±5 dB(A), which was repeated 10 times. The volume was measured using a sound level meter (Voltcraft SL-50, Hirschau, Germany).

### Statistics

Intra-individual averages of 10 repetitions were initially calculated for each subject and each parameter. Mean values and standard deviations were calculated for all parameters and each sound for both groups (patients and healthy volunteers). An unpaired t-test was used to reveal differences between the two groups for the different sounds. The statistical analysis of all collected data was performed using SPSS (version 24.0; IBM Corp, Armonk, NY). A p-value <5% (p<0.05) was considered significant.

## Results

**Figure 2:**
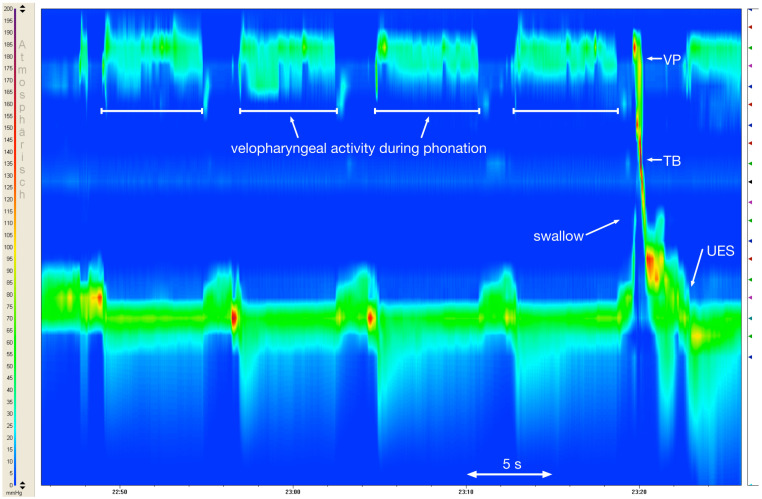
High resolution manometry clouse contour plot showing the pressures (color coded 0 mmHg–200 mmHg) in the velopharynx (VP) and upper esophageal sphincter (UES) during the repeated articulation of the sound /i:/ (TB=tongue base)

HRM data of patients as well as healthy participants was recorded without any significant problems. Figure 2 (Fig. 2) demonstrates repeated articulations of the sound /i:/ in the HRM contour plot. 

**Figure 3:**
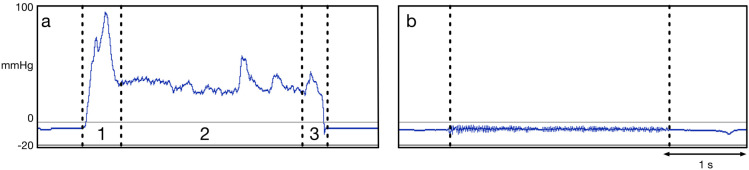
Comparison of the 3-phase-model (indicated as 1, 2, 3) of the articulation of /i:/ by a healthy subject (a) and a patient with UCLP (b), where the blue curve demonstrates the pressure (mmHg) in the velopharynx over time

Both groups presented with similar pressure curves in the velopharynx for each phoneme with regards to the phases described and pressure peaks, but differed in the total pressure values. An exception was noted for the sound /i:/, where a 3-phase-model could not be seen for most patients with UCLP due to a reduced pressure build-up (Figure 3 (Fig. 3)). As expected, the three phases as previously described by Jungheim et al. [36] could also not be seen during the articulation of the alveolar nasal sound (/n/) in both groups as there was no relevant pressure build-up.

Mean values and standard deviations for both groups and all measured parameters are shown in Table 1 (Tab. 1). Highest mean values for P_max1_, P_max2_ and P_max3_ as well as average pressures (P_Ø_) were found in group 1 for all tested sounds except the nasal. These differences showed statistical significance for sound number 2 (/s/), the alveolar sibilant (Table 1 (Tab. 1)) (P_max1_ p=0.002, P_max2_ p=0.007, P_max3_ p=0.005, P_Ø_ p=0.008). An extended duration of t_1_ and t_3_ was not assignable to one of the groups, but varied (see Table 1 (Tab. 1)). Slope values for both groups were fairly similar and showed values of around 1 for either group, indicating no significant pressure change during sustained sound production. Only for sound 2 (/s/) group 1 showed a pressure increase during the plateau phase, where group 2 dropped in pressure.

## Discussion

Very little is known about the pressures in the velopharyngeal area during the production of sounds. A 3-phase-model has previously been described for the pressure curve of the velopharynx during phonation [36]. As part of this investigation, this model could be verified for different sound groups in healthy individuals, but the model cannot be applied to all pressure curves in patients with UCLP. In particular, the production of the tested vowel cannot be adequately described with the existing model (Figure 1 (Fig. 1), Figure 3 (Fig. 3)), so that further models need to be developed. 

As assumed, the tested parameters did reveal higher maximum and average pressures for the oral sounds in the group of healthy volunteers. Even though statistical significances were only found in relation with the alveolar sibilant, pressure differences between both groups are rather large for all the tested sounds (with exception of the nasal sound), where group 2 in all instances only measures up to half of the pressure values recorded for group 1 (see Table 1 (Tab. 1)). The lack of statistical significance might be explained by the large standard deviations present especially during vowel production. 

As hypothesized, reduced velopharyngeal pressures during speech sound production can be confirmed for patients with UCLP and it can be assumed that reduced pressures contribute to the development of hypernasality.

It has previously been reported that nasal emission is rarely noticeable during the production of vowels due to the physiological lack of a pressure build-up and release [6]. Even though one might conclude that with the laminar air flow which is present in vowels, it would also be easier for patients with UCLP to maintain a stronger pressure and tighter closure in the velum [17]. Values of this study show that the actual difference in mean maximum pressures is very similar to the pressure difference in the other tested sounds, where group 2 reaches only less than half the pressure values of group 1. These values indicate that the underlying anatomical differences between the tested groups result in differences in velopharynx functioning even in non-complex sounds. 

Reports of nasalance measurements, which quantify the sound energy during articulation, have suggested differences in nasalance levels for different sounds. In healthy individuals an increased nasalance has been found for high front vowels [41]. Higher sound energy profiles could in theory be caused by lower pressures in the velopharyngeal area. The study described here showed that total pressures of the high front vowel were indeed lower than total pressures of the alveolar sibilant for either group. However, the values are very similar to the ones measured for postalveolar sibilant production. In order to address this postulate, total pressures of the high front vowel /i/ would need to be compared to pressure profiles in relation with different vowel productions, especially back and low vowels. However, as this study set-up did not include other vowels, this question cannot be answered. Whether the higher nasalance measured in relation with high front vowels [41] might also relate to the fact that the pressure profile of the vowel /i/ production does not comply with the existing pressure model [36], cannot be answered by the data collected (here) and needs to be part of further studies. 

Fricatives fall into the category of pressure-sensitive phonemes [6], which means that they are most likely to show nasal air emission due to the required build-up and release of intraoral pressure [6]. The reduced maximum pressures of patients with UCLP in comparison to healthy subjects also indicate that due to this “less tight” closure, some nasal emission results and with that, despite of the existence of a velopharyngeal “closure” in terms of recorded pressures, hypernasality can still be existent. Consequently, in the patients examined here the velopharyngeal insufficiency was represented by a reduced velopharyngeal occlusion pressure. 

It is also interesting that a statistically relevant difference in pressure profiles and maximum pressures between patients with UCLP and healthy subjects only exists for the speech production of the alveolar sibilant and not the postalveolar one, even though those two sounds both are complex sounds which require a tight closure of the velum and present with the required buildup and release of intraoral air pressure [17]. Kuehn et al. [22] reported also that the lingua-dorsal consonant and lingua-apical consonant may differ in velopharynx pressures in healthy individuals. As the group investigated varying phonetic contexts and this study focused on the isolated and sustained phonation, results are only partially comparable. A reason for the difference might lie in the different articulation zone for both sounds. With the postalveolar placement of the friction, the tongue is retracted further than for the alveolar sibilant. By this a compensatory mechanism might be supported in which the back of the tongue supports the velar elevation and contact, which is represented by more similar pressures among both groups especially during the steady phase (Table 1 (Tab. 1)). Compensatory strategies during the production of sibilants, such as change of place of articulation for instance, by subjects with severe hypernasality have been reported in the literature before [6]. 

As the passage between the nasopharynx and oropharynx is open for the articulation of nasal sounds and no pressures are recordable (also see [22] for comparison), as expected, certain parameters were not extractable for sound number 4, the alveolar nasal sound, in both groups. In a state of rest velopharyngeal functions between both groups do not differ. 

The question arises how much pressure is actually necessary during articulation of certain sounds to ensure the velopharyngeal closure is tight enough to avoid nasal resonance and hypernasality. All of the patients with UCLP participating in this study were diagnosed with hypernasality. Even though mean pressure values between the two tested groups did not reach statistical significance for all the speech sounds tested, the actual mean values between the groups differ strongly. It can be concluded that the pressure profiles of patients with UCLP are not sufficient to eliminate nasal resonance during sound production especially for more complex sounds. These results help to give a general understanding of hypernasality during speech, but a more detailed investigation will be necessary to take into account the degrees of nasality resulting from certain velopharyngeal pressures. 

High resolution manometry represents an established tool in gastroenterology to visualize the peristalsis in the esophagus. In recent years it has become more and more recognized in phoniatrics and speech pathology in order to investigate closing pressures of the pharynx during swallowing as well as speech. As there are many indirect procedures for measuring velopharyngeal function during speech, it is capable of providing valuable information by directly measuring velopharyngeal closure pressures exactly where they happen. Especially with regards to children, established tools will be continued to be used for the investigation of nasalance. However, HRM represents a very precise tool in the investigation of rhinophonia, as it is able to measure pressures directly at the velopharyngeal closure, allowing conclusions about the velopharyngeal function even though a slight invasiveness during the placement of the probe needs to be accepted. 

## Notes

### First authorship

Simone Miller and Johanna Kallusky share first authorship.

### Authors’ ORCIDs

- Dr. rer. biol. hum. Simone Miller: 0000-0002-5289-1795
- PD Dr. med. Dr. med. dent. Rüdiger Zimmerer: 0000-0002-1859-4324
- Prof. Dr. med. Dr. med. dent. Frank Tavassol: 0000-0003-0975-7467
- Prof. Dr. med. Dr. med. dent Nils-Claudius Gellrich: 0000-0002-8800-4596
- Prof. Dr. med. Dr. med. h.c. Martin Ptok: 0000-0001-6296-1323
- Prof. Dr. med. Michael Jungheim: 0000-0003-4823-3746

### Ethics approval

The study was performed in accordance with the Declaration of Helsinki, Good Clinical Practices, and applicable regulatory requirements. The clinical study was approved by the institution’s ethics committee (number #5902/2011). 

### Consent to participate

All participants signed an informed consent form before undergoing any study-related procedures and were not financially remunerated.

### Competing interests

The authors declare that they have no competing interests.

## Figures and Tables

**Table 1:**
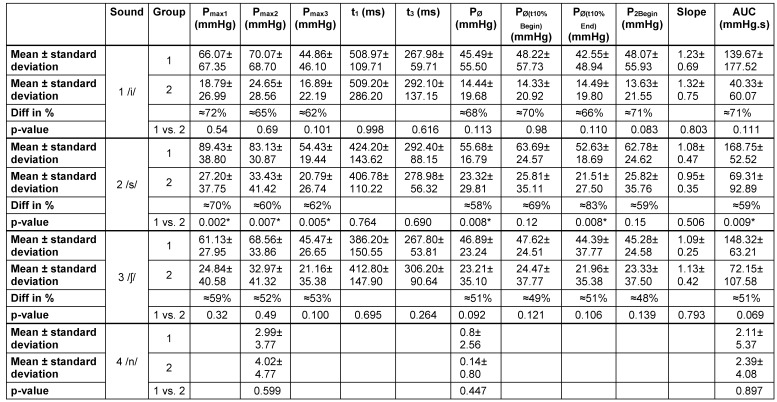
Mean values and standard deviations of all parameters for both groups (group 1: healthy individuals, group 2: patients with UCLP) and sound (1: /i:/ 2: /s/ 3: /ʃ/ 4: /n/). Mean differences of group comparison (t-test for independent samples); p-values of p<0.05 represent statistical significance.
